# Conjugate Meningococcal Vaccines Development: GSK Biologicals Experience

**DOI:** 10.4061/2011/846756

**Published:** 2011-07-18

**Authors:** Jacqueline M. Miller, Narcisa Mesaros, Marie Van Der Wielen, Yaela Baine

**Affiliations:** ^1^GlaxoSmithKline Biologicals, 2301 Renaissance Boulevard, King of Prussia, PA 19406, USA; ^2^GlaxoSmithKline Biologicals, Avenue Fleming 20 (W23), 1300 Wavre, Belgium

## Abstract

Meningococcal diseases are serious threats to global health, and new vaccines specifically tailored to meet the age-related needs of various geographical areas are required. This paper focuses on the meningococcal conjugate vaccines developed by GSK Biologicals. Two combined conjugate vaccines were developed to help protect infants and young children in countries where the incidence of meningococcal serogroup C or serogroup C and Y disease is important: Hib-MenC-TT vaccine, which offers protection against *Haemophilus influenzae* type b and *Neisseria meningitidis* serogroup C diseases, is approved in several countries; and Hib-MenCY-TT vaccine, which adds *N. meningitidis *serogroup Y antigen, is currently in the final stages of development. Additionally, a tetravalent conjugate vaccine (MenACWY-TT) designed to help protect against four meningococcal serogroups is presently being evaluated for global use in all age groups. All of these vaccines were shown to be highly immunogenic and to have clinically acceptable safety profiles.

## 1. Introduction

Invasive diseases caused by *Neisseria meningitidis*, of which meningitis and septicaemia are the most important, are serious threats to global health [1, 2]. Sporadic as well as endemic cases occur worldwide, and *N. meningitidis* is the only encapsulated bacterium known to cause large epidemics of bacterial meningitis [3, 4]. Notably, extensive meningococcal disease outbreaks comprising hundreds of thousands of cases occur cyclically in an area of Sub-Saharan Africa, also called the Meningitis Belt [5–8]. Overall, about 500,000 cases of meningococcal disease occur each year causing at least 50,000 deaths [9]. Meningococcal meningitis has a case-fatality rate of 5% to 10% in industrialised countries, which can reach 20% in the developing world [10, 11]. In addition, 12% to 19% of survivors develop long-term neurological sequelae [3, 7, 12–14]. While the highest case-fatality rate is observed among persons older than 65 years and generally decreases with lower age [10], the risk of meningococcal disease is highest in infants and young children with a secondary peak in incidence during adolescence and young adulthood [15].

*N. meningitidis* is a gram-negative encapsulated diplococcus that colonises the human nasopharynx, where it is usually carried asymptomatically [1]. Meningococci are transmitted through close contact via respiratory droplets [7]. In some cases, bacteria spread from the nasopharynx to nearby epithelial cells causing local invasion of tissue. If the meningococci reach the bloodstream, they may cause meningococcal meningitis or fulminant septicaemia [3, 7, 16]. *N. meningitidis* is classified into 13 serogroups according to differences in the capsular polysaccharide (PS) antigens. Six of these serogroups (A, B, C, W-135, Y, and more recently X) are responsible for the majority of meningococcal disease cases [3, 17].

Meningococcal incidence and serogroup distribution are highly regional and have a cyclical nature, with peaks typically occurring in a five-to-eight-year pattern [18, 19]. For this reason, meningococcal disease surveillance is required for the assessment of local epidemiology and disease burden, which are key issues for vaccine formulation and prevention strategies [19]. Although many of the surveillance systems for meningococcal disease lack sensitivity and may underestimate disease burden, current meningococcal disease epidemiology can be summarised per region [19]. In Africa and Asia, serogroup A (MenA) remains the cause of most large-scale epidemics, with the highest magnitude in the African Meningitis Belt, while serogroups B and C (MenB and MenC) are associated with sporadic disease [3, 19–21]. In addition, serogroup W-135 (MenW-135) has emerged as a new threat after causing outbreaks in Hajj pilgrims in Saudi Arabia, followed by Burkina Faso and Chad [22, 23]. More recently, various outbreaks due to serogroup X have also been reported in Africa [24–26]. In industrialised countries such as Europe, the United States (USA), Latin America and Australia, MenB and MenC are the most important causes of invasive meningococcal disease [2, 14, 19, 27–29]. In addition, serogroup Y (MenY) accounts for around one-third of meningococcal disease cases in the US and the incidence of this serogroup has also recently increased in Scandinavian countries [10, 14, 17, 30]. In industrialised countries, rates of meningococcal disease are presently very low (0.5–6 per 100,000 population) and this may be explained by a combination of environmental, organism and host factors. Even with this historically low rate, meningococcal disease continues to cause considerable morbidity and mortality among all age groups in these regions and *N. meningitidis* remains the most common cause of bacterial meningitis in children and young adults [2, 10].

While mass chemoprophylaxis is not recommended to control large outbreaks of meningococcal disease, vaccination is considered to be an effective prevention strategy and the development of effective meningococcal vaccines, which have acceptable safety profiles, is a public health priority [31, 32]. The first vaccines developed were plain PS vaccines that consist of purified capsular PS from specific meningococcal serogroups. GlaxoSmithKline (GSK) Biologicals developed different formulations of plain PS vaccines against serogroups A, C, W-135, and Y (*MencevaxAC*, *MencevaxACW*, *MencevaxACWY*). These vaccines have been used to immunise travellers, Hajj pilgrims, military personnel, and other specific populations at increased risk for invasive meningococcal disease [33, 34]. Meningococcal PS vaccines are available for use in children above two years of age but, aside from the PS from MenA, which is immunogenic in infants as young as three months of age, they are suboptimally immunogenic and therefore not indicated in infants and toddlers [35, 36]. Moreover, PS vaccines induce T-cell-independent immune responses, which are not longlasting, do not result in immune memory and can induce immunological hyporesponsiveness for serogroups A, C, W-135, and Y. For these reasons, PS vaccines are not appropriate when sustained protection is needed [37–40].

To overcome these limitations, conjugate vaccines were developed, in which capsular PS are covalently coupled to carrier proteins that contain T-cell epitopes. These vaccines have been shown to be immunogenic in infants, T-cell dependent, induce immune memory, and induce higher bactericidal titres, which potentially could translate into longer antibody persistence [37, 38, 41]. The most common carrier proteins used in conjugate meningococcal vaccines are tetanus toxoid (TT), diphtheria toxoid (DT), and a nontoxic cross-reacting mutant of DT (CRM_197_) [38]. Conjugate vaccines against MenC were introduced in 1999 in the United Kingdom (UK) as part of a vaccination programme that included routine infant immunisation and a large-scale catch-up campaign from infants through children 18 years of age, which was later extended to 24 years of age. This programme had a tremendous impact on the incidence of the disease and induced herd immunity [42–44]. MenC vaccination programmes have been successfully implemented in other countries as well, including Canada, Spain, and the Netherlands [45–47]. Presently, two tetravalent conjugate vaccines against serogroups A, C, W-135, and Y, using DT or CRM_197_ as carrier proteins, have been licensed in various countries.

Unlike other meningococcal serogroups (A, C, W-135, and Y), capsular PS from MenB is poorly immunogenic in humans. Furthermore, the capsular structure of MenB mimics sialylated structures in human neural tissue posing potential safety concerns for a vaccine formulation containing these polymers. Therefore, most of the MenB vaccine research has been focused on the outer membrane proteins (OMPs) [48]. MenB vaccines have been developed using alternative strategies and they are available in Cuba, New Zealand, the Netherlands, and France [29]. However, these vaccines are strain specific and therefore their use is limited [49]. A protein-based investigational MenB vaccine intended for global use has been developed by Novartis, and a Marketing Authorization Application was submitted to the European Medicines Agency (EMA) at the end of 2010. A second investigational protein-based MenB vaccine being developed by Pfizer is currently in Phase II of clinical development. 

In this paper, we will focus on vaccines, developed by GSK Biologicals, against the most common serogroups for which PS conjugate vaccine technology can be used (MenA, MenC, MenW-135, and MenY). The vaccine development strategy was driven by the populations at highest risk: first the developing world with a focus on Africa, then more industrialised countries with a focus on the highest risk populations (infants and young children, followed by adolescents). These vaccines have been studied in randomised, controlled clinical trials evaluating the immunogenicity, antibody persistence, induction of immune memory, reactogenicity, and safety of the different vaccines. Results of the studies that are discussed here have been previously presented in peer-reviewed publications (Table 1).

## 2. Conjugate Meningococcal Vaccines Developed by GSK Biologicals

The effectiveness of meningococcal vaccines is challenging to evaluate in prospective, randomised efficacy studies because the incidence of endemic meningococcal disease is low, and epidemics are difficult to predict. Therefore, efficacy is inferred from immunogenicity data via laboratory markers that can reliably predict clinical protection [50]. The primary human defence to meningococcus is the activation of the complement system by antigen-specific antibodies resulting in bacterial lysis. Bactericidal assays measuring interactions of antibodies and human complement at the bacterial surface (hSBA) can be used to measure the functional activity of a tested serum against a given strain of meningococcus. Data generated in US military recruits suggested that an hSBA titre ≥1 : 4 correlated with protection against meningococcal serogroup C disease [51]. After introduction of MenC conjugate vaccines in the UK another serological correlate of protection based on a serum bactericidal assay using rabbit complement (rSBA titre ≥1 : 8) became available using both prelicensure age-stratified seroprevalence and disease incidence data, and postlicensure efficacy estimates [52–54]. The seroprotective thresholds for MenC have been extended to serogroups A, W-135, and Y. Both assays are accepted by the WHO for the assessment of the immunogenicity of meningococcal vaccines [55] and both have been used in GSK's clinical development depending on regional health authority requirements.

### 2.1. DTPw-HBV/Hib-MenAC Combined Vaccine for African Infants

In Africa, the world's highest risk population, PS vaccines against meningococcal disease are available but they do not offer broad serogroup protection to infants and in older subjects they only protect for 3–5 years, leaving the population vulnerable to future epidemics [56–58]. Moreover, the hyporesponsiveness that may be induced by repeated injections of PS vaccines compromises their use in the future because the population remains at life-long risk for invasive meningococcal disease [37].

In 2000, the WHO and representatives from eight African countries called for the development of new conjugate vaccines, which could be used in routine vaccination campaigns to control invasive meningococcal disease in Africa. In response to this request, GSK Biologicals initiated the development of a new heptavalent diphtheria, tetanus, whole cell pertussis, hepatitis B, *Haemophilus influenzae* type b, *N. meningitidis* serogroups A, and C- tetanus toxoid conjugate combined vaccine (DTPw-HBV/Hib-MenAC-TT) intended for vaccination of infants [59–61]. Indeed, MenA and to a lesser extent MenC contribute to endemic disease and periodic outbreaks in Africa and at that time, MenW-135 was not yet recognised as an important serogroup in the African Meningitis Belt [22, 23]. The addition of antigens from MenA and MenC to antigens of vaccines routinely administered in paediatric vaccination programmes was intended to promote rapid uptake and high coverage of these components without the need for further vaccination visits or injections, while minimising costs [62–64].

Three-dose primary vaccination with DTPw-HBV/Hib-MenAC-TT was shown to be noninferior to DTPw-HBV/Hib for the five common antigens and induced antibodies against MenA and MenC in the majority of subjects when given to African infants at 6, 10, and 14 weeks of age [60]. The vaccine induced a good persistence of antibodies and immune memory [59]. As for other conjugate vaccines used in infancy [52, 65, 66], a booster dose was required in the second year of life to sustain protection through early childhood [60]. The DTPw-HBV/Hib-MenAC-TT vaccine had a clinically acceptable safety profile and no specific safety issues were raised during the Phase II and Phase III studies [59–61]. No increase in reactogenicity during the primary vaccination course was observed when compared with the licensed DTPw-HBV/Hib vaccines [59].

Following development of this new vaccine, the WHO communicated that they were prioritising mass vaccination in older individuals and for MenA only, since this is currently the predominant serogroup in the African Meningitis Belt. As DTPw-HBV/Hib-MenAC-TT was a combination vaccine designed to be incorporated into the routine vaccination schedule for younger children, final development of the vaccine was stopped following consultation with the WHO. Recently, a new MenA conjugate vaccine (*MenAfriVac*, the Serum Institute of India) was licensed and prequalified by the WHO. However, this new vaccine will only provide protection against MenA and there may be need in the future for conjugate vaccines directed against other serogroups.

### 2.2. Hib-MenC-TT Combined Vaccine for Infants: *Menitorix*

The next high-risk population targeted by GSK Biologicals for the development of conjugate vaccines were infants and young children in countries where MenC is the predominant serogroup, since these age groups are at the highest risk for invasive disease in most countries worldwide [2, 10, 19]. As the list of recommended vaccines to be included in childhood immunisation programmes increases, combination vaccines have been recognised as a means of simplifying immunisation schedules [62–64]. Therefore, GSK Biologicals initiated the development of a combined vaccine (Hib-MenC-TT) intended to provide protection to infants and toddlers against two major pathogens responsible for bacterial meningitis (*H. influenzae* type b [Hib] and *N. meningitidis* serogroup C [MenC]). The Hib-MenC-TT vaccine offers an alternative vaccination schedule to the acellular pertussis (DTPa)-Hib combinations coadministered with standalone MenC conjugate vaccines [67].

Hib-MenC-TT contains 5 *μ*g polyribosylribitol phosphate (PRP) from Hib and 5 *μ*g PS from MenC, each conjugated to TT. Phase II and Phase III studies have shown that the Hib-MenC-TT vaccine is immunogenic when administered as three-dose primary series in infants at 2, 3, and 4 months of age [68–70] or at 2, 4, and 6 months of age [62, 71] (Table 2). At least 98.8–100% of subjects had anti-PRP ≥0.15 *μ*g/mL (data not shown) and 99.2–100% had rSBA-MenC titres ≥1 : 8 (Table 2) after completion of the primary vaccination schedule. The Hib-MenC-TT combined vaccine induced MenC and Hib responses comparable to those induced by licensed monovalent vaccines and all primary hypotheses regarding the noninferiority of Hib-MenC-TT to licensed Hib and monovalent MenC conjugate were met [62, 68, 70, 72]. Consistently high anti-PRP GMCs were observed across the studies, suggesting that Hib-MenC-TT can overcome the challenges seen with reduced Hib immunogenicity when Hib conjugate vaccines are given in combination with acellular pertussis compared with separate injections [67].

Moreover, primary vaccination with Hib-MenC-TT induced persistent antibodies against both antigens up to the second year of life [68, 70, 73, 74]. The immune response postvaccination and the persistence of anti-PRP antibodies up to the booster vaccination was higher following vaccination with Hib-MenC-TT than following vaccination with DTPa/Hib combined vaccines [73, 74]. Moreover, the persistence of rSBA-MenC antibodies, when measured before booster vaccination, was higher in subjects primed with Hib-MenC-TT than in subjects primed with MenC-CRM_197_ although lower than in subjects primed with MenC-TT [73, 74].

The booster dose administered in the second year of life resulted in very high seroprotection rates against both Hib and MenC [68, 71, 73–75] (Table 2). After the booster vaccination, the percentages of Hib-MenC-TT primed children with anti-PRP ≥1.0 *μ*g/mL and rSBA-MenC titres ≥1 : 128 were 100% (data not shown) and 97.4–100% (Table 2), respectively. Long-term persistence of the immune response induced by the booster was observed up to two years after vaccination [76]. The totality of the clinical data collected demonstrated that Hib-MenC-TT can be used as booster dose for toddlers previously primed with MenC and Hib in infancy [72–74]. Of note, the noninferiority of Hib-MenC-TT to monovalent Hib and MenC conjugate vaccines was demonstrated when administered as a single dose in Hib-primed but MenC-naïve toddlers [72]. The evaluation of the persistence of the Hib and MenC immunogenicity after different vaccination schedules is ongoing for up to five years postbooster vaccination.

In addition, one booster dose of Hib-MenC-TT, given to children aged 6–12 years who were primed six years before with MenC-CRM_197_, was shown to be highly effective and rSBA-MenC antibody titres ≥1 : 8 were sustained for at least one year in 99.6% of the vaccines [77]. However, these results were somewhat contradictory with other studies showing reduced postbooster responses and antibody persistence when MenC-CRM_197_ was used for priming, regardless of whether a CRM_197_ or a TT conjugate vaccine was used for boosting [78, 79].

Notably, no evidence of negative interference on the immune response to any of the antigens was observed when the Hib-MenC-TT vaccine was coadministered with common childhood vaccines including combined measles, mumps, and rubella vaccine [MMR], combined diphtheria, tetanus, acellular pertussis, and inactivated polio vaccine [DTPa-IPV], a combined diphtheria-tetanus-acellular pertussis-hepatitis B-inactivated poliovirus vaccine [DTPa-HBV-IPV], a 7-valent pneumococcal conjugate vaccine (7vCRM) and a 10-valent pneumococcal nontypeable *H. influenzae* protein D-conjugate vaccine [PHiD-CV] [62, 69, 71, 75, 80].

In reported studies, a total of 3127 children less than two years were vaccinated with Hib-MenC-TT combined vaccine. In primary and booster studies, Hib-MenC-TT was comparable to control monovalent Hib and MenC vaccines in term of local reactogenicity (pain, redness, and swelling) and general solicited symptoms (fever, irritability/fussiness, drowsiness, and loss of appetite), with some evidence of lower rates of fever. The clinical studies supported the acceptability of the safety profile of Hib-MenC-TT.

In the UK, Hib-MenC-TT has been used as booster vaccination for the Hib and MenC antigens in the second year of life (after monovalent MenC and DTPa/Hib priming in infants) since September 2006. The vaccine is currently approved in some EU countries, in Brazil, and in New Zealand for three-dose primary vaccination in infants from the age of two months up to 12 months and for booster vaccination in toddlers up to the age of two years. The vaccine is also approved in Australia for one-dose vaccination in toddlers previously primed with Hib but naïve for MenC [72].

In summary, Hib-MenC-TT was shown to be immunogenic with an acceptable safety profile when given to infants and toddlers. Primary vaccination resulted in immune priming and the induction of immune memory as reflected by antibody persistence and response to booster vaccination. The consistent observation of significantly higher anti-PRP GMCs versus control vaccines suggests that placing the Hib antigen in combination with meningococcal antigens can overcome the reduction in Hib immunogenicity noted in combination vaccines including Hib and acellular pertussis antigens [67].

### 2.3. Investigational Hib-MenCY-TT Combined Vaccine for Infants

Unlike in most other industrialised countries, MenY is also a major cause of meningococcal disease in the USA, where MenC and MenY together account for approximately two-thirds of invasive disease [10, 92]. In addition, this serogroup has more recently also been noticed in other regions, including Europe [30]. For this reason, GSK Biologicals is developing an investigational combination vaccine (Hib-MenCY-TT) designed to protect infants and toddlers against invasive diseases caused by MenC, MenY, and Hib. This vaccine has been investigated according to a four-dose immunisation series at 2, 4, 6, and 12–15 months or 2, 3, 4, and 12–18 months. The 2, 4, 6, and 12–15 months schedule was studied because this is the currently recommended Advisory Committee on Immunization Practices schedule for routine Hib conjugate in the USA. Serogroup Y has been included in the investigational combination vaccine because serogroup Y causes approximately 1/3 of invasive meningococcal disease in the USA [10].

A single dose of Hib-MenCY-TT contains 2.5 *μ*g PRP from Hib and 5 *μ*g each of PS from MenC and MenY, all conjugated to TT. In Phase II studies, Hib-MenCY-TT was shown to be immunogenic for all three antigens after three-dose vaccination at 2, 3, and 4 months [68] or 2, 4, and 6 months [81, 82, 84] of age and after a fourth dose at 12–18 months of age [68, 83, 84] (Tables 3 and 4). All primary hypotheses regarding the noninferiority of Hib-MenCY-TT to licensed Hib and monovalent MenC conjugate vaccines were met, and as with the Hib-MenC-TT vaccine, consistently higher anti-PRP concentrations were observed with Hib-MenCY-TT than with licensed Hib conjugate vaccines [68, 82, 84]. In addition, vaccination of infants with Hib-MenCY-TT induced an immune response against MenC and MenY that was significantly higher than that induced by one injection of a licensed tetravalent polysaccharide vaccine in older children [82]. The antibody persistence of the fourth dose was shown for at least one year [83].

More recently, a Phase III study conducted in the USA, Mexico, and Australia confirmed that Hib-MenCY-TT was highly immunogenic to the meningococcal antigens and that the immune response induced by this vaccine in terms of anti-PRP concentrations was noninferior to that induced by licensed Hib vaccines when used according to the 2, 4, 6, and 12–15 months schedule [85].

Clinical data for the investigational Hib-MenCY-TT vaccine did not indicate interference with common childhood vaccines including DTPa-HBV-IPV, 7vCRM, MMR, and a varicella vaccine [81, 82]. The lack of immune interference with these antigens that are established in paediatric vaccination schedules is critical for the successful incorporation of the novel antigens. 

The reactogenicity and safety profile of Hib-MenCY-TT was shown to be clinically acceptable in infants and toddlers who received four consecutive doses of this vaccine coadministered with routinely recommended paediatric vaccines. The reactogenicity profile of Hib-MenCY-TT was similar to that of Hib-TT, Hib-OMP and MenC-CRM_197_ and the incidence of grade 3 symptoms was low in all the studies [68, 81–85].

In summary, Hib-MenCY-TT was shown to be immunogenic to the Hib and the meningococcal serogroup C and Y components when given to infants and toddlers as four-dose immunisation series. Hib-MenCY-TT was noninferior to licensed monovalent control vaccines and demonstrated a clinically acceptable safety profile. An application for licensure of this vaccine was submitted to the US Food and Drug Administration (FDA) on August 12, 2009 and is currently under review. Hib-MenCY-TT is not currently licensed in any country.

### 2.4. Investigational MenACWY-TT Conjugate Vaccine for All Age Groups

Besides infants and young children, adolescents are the second key age group at increased risk for meningococcal disease. Accordingly, GSK Biologicals is developing a new investigational tetravalent meningococcal conjugate vaccine with PS from serogroups A, C, W-135, and Y conjugated to TT (MenACWY-TT) to complement the two other tetravalent conjugate vaccines that are currently available. Indeed, as previously discussed, primary vaccination with a MenC-TT vaccine has been demonstrated to result in higher rSBA-MenC titres as compared to priming with either of the two monovalent MenC conjugate vaccines that use CRM_197_ as carrier protein, translating into higher persistence up to one year after booster, regardless of the booster vaccine used [78, 79, 93]. Unlike Hib-MenC-TT and Hib-MenCY-TT, MenACWY-TT is not only intended for vaccination of infants and toddlers and therefore Hib was not included as a component of the vaccine. Although the MenACWY-TT vaccine was developed with a focus on adolescents, the investigational vaccine is being evaluated in various age groups including infants (NCT01144663).

**Figure 1:**
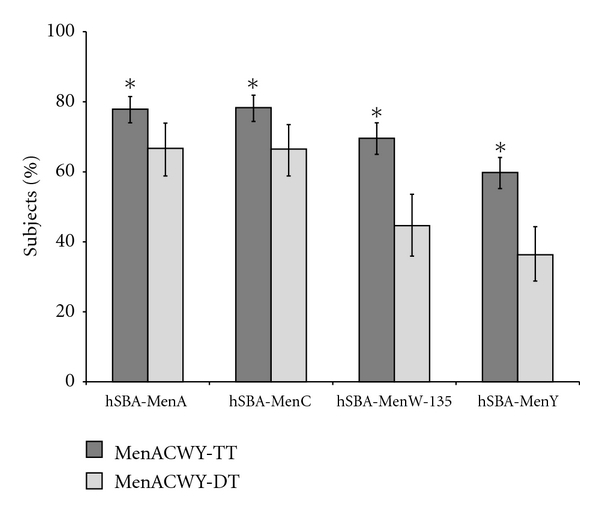
Vaccine response for hSBA antibodies one month after vaccination with MenACWY-TT or with a control vaccine in subjects aged 11–25 years (ATP cohort for immunogenicity). (MenACWY-TT: subjects vaccinated with MenACWY-TT, MenACWY-DT: subjects vaccinated with MenACWY-DT, vaccine response defined as (1) for initially seronegative subjects, antibody titres ≥1 : 16 at one month after vaccination, (2) for initially seropositive subjects, antibody titres at one month after vaccination ≥4-fold the prevaccination antibody titre. Error bars represent the 95% confidence interval. *Statistically significantly higher value in the ACWY-TT group compared to the ACWY-DT group (exploratory analysis). Results of this figure are currently in publication [89].)

Phase II and phase III studies showed that a single dose of MenACWY-TT was highly immunogenic in toddlers, children, adolescents and adults at one month following vaccination (Tables 5, 6 and 7). The observed rSBA and hSBA GMTs against MenC were significantly higher following MenACWY-TT vaccination in toddlers in the second year of life when compared to vaccination with a licensed serogroup C conjugate vaccine (MenC-CRM_197_) [87, 90, 91]. Recent studies have shown that MenACWY-TT did not interfere with DTPa-HBV-IPV/Hib or a combined MMR and varicella vaccine (MMRV) in toddlers, nor did it affect the safety profile of these vaccines [90, 91]. In children [87], adolescents [88], and young adults [86], exploratory analyses showed that MenACWY-TT induced statistically significantly higher rSBA GMTs for all serogroups compared to a licensed tetravalent plain PS vaccine. In the study conducted in subjects aged 11–17 years, noninferiority of MenACWY-TT to the licensed tetravalent PS vaccine was demonstrated in terms of percentage of subjects with a vaccine response [88]. In addition, a recent study showed that the proportion of subjects with a vaccine response and the hSBA GMTs were higher after vaccination with MenACWY-TT than with a licensed tetravalent diphtheria toxoid conjugate vaccine (MenACWY-DT) (Figure 1) [89].

Overall, MenACWY-TT was shown to have increased reactogenicity as compared to plain PS vaccines in children, adolescents, and adults and to have a similar reactogenicity profile compared to conjugate vaccines: MenC-CRM_197_ in toddlers younger than two years and MenACWY-DT in adolescents [86–91]. These results were expected as conjugate vaccines are known to be more reactogenic than PS vaccines and similar findings were observed with a PS vaccine compared to MenACWY-DT and MenACWY-CRM_197_ [94, 95]. Given the relatively infrequent reports of grade 3 symptoms (≤9.3% of subjects [87]), the MenACWY-TT vaccine was considered to have an acceptable safety profile.

In summary, a single dose of MenACWY-TT was shown to be highly immunogenic for serogroups A, C, W-135, and Y in toddlers, children, adolescents, and young adults and had a clinically acceptable safety profile. A Marketing Authorization Application was submitted to the European Medicines Agency (EMA) on March 4, 2011 and is currently under review. MenACWY-TT is not currently licensed in any country.

## 3. Conclusions and Perspectives

Because of the severity of invasive meningococcal disease, the rapidity with which it develops, and the high frequency of long-term sequelae, development of effective meningococcal vaccines with clinically acceptable safety profiles is a public health priority. The complete portfolio of conjugate meningococcal vaccines developed by GSK Biologicals is designed to respond to the global need with prioritisation in infants and young children, followed by adolescents, the age groups at highest risk. Two vaccines, using TT as carrier protein, were designed to help protect infants and young children against meningococcal serogroup(s) C (and Y) and Hib diseases in countries where these serogroups are major contributors to meningococcal epidemiology. The first vaccine (Hib-MenC-TT) is presently approved in various countries worldwide for use as three-dose primary vaccination in infants and booster vaccination in toddlers or as one-dose vaccination in toddlers previously primed with Hib but naïve for MenC. Primary vaccination resulted in immune priming and induced immune memory as reflected by antibody persistence and response to booster vaccination. The second vaccine (Hib-MenCY-TT) adds serogroup Y antigen and an application for licensure of this vaccine as four-dose immunisation series in infants and toddlers is currently under review by the US FDA. All primary hypotheses regarding the noninferiority of both vaccines to licensed Hib (DTPa/Hib combinations or Hib standalone vaccines) and monovalent MenC conjugate vaccines were met. On the other hand, GSK Biologicals has more recently evaluated an investigational tetravalent conjugate meningococcal vaccine (MenACWY-TT) designed to offer protection against four of the six most common serogroups worldwide for use in all age groups. This vaccine was shown to be highly immunogenic in toddlers, children, adolescents, and adults. All these vaccines were shown to have an acceptable safety profile, which was comparable to licensed conjugate vaccines. As demonstrated by the broad portfolio of meningococcal conjugate vaccines, GSK remains committed to the development of meningococcal vaccines, targeting the populations at greatest risk.

##  Conflict of Interests

J. Miller, N. Mesaros, M. V. D. Wielen, and Y. Baine are employees of GlaxoSmithKline Biologicals. Drs M. Miller, N. Mesaros, M. V. D. Wielen, and Y. Baine report ownership of stock options.

## Figures and Tables

**Table 1:** Published clinical vaccination trials of GSK's conjugate meningococcal vaccines.

Vaccine	Study	Phase	Country	Target group	Schedule	*N*	Publication
	NCT00317174	Phase II	Philippines	Infants	6, 10, 14 weeks PS challenge at 10 months	524 217	Gatchalian et al., 2008 [59]*
DTPw-HBV/Hib-MenAC	ISRCTN35754083	Phase II	Ghana	Infants	6, 10, 14 weeks PS challenge at 12 months	280 260	Hodgson et al., 2008 [60]
	NCT00317161 and NCT00317187	Phase III	Philippines, Thailand	Infants	2, 4, 6 months	1780	Kerdpanich et al., 2008 [61]

	NCT00135486 and NCT00135564	Phase II	Germany	Infants	2, 3, 4 months PS challenge at 12–15 months	520	Schmitt et al., 2007 [70]
	NCT00323050 and NCT00322335	Phase III	Spain	Infants	2, 4, 6 months 13-14 months	237 358	Tejedor et al., 2007 [62] Tejedor et al., 2008 [74]
Hib-MenC-TT	NCT00263653	Phase III	Spain	Toddlers	13-14 months	297	Carmona et al., 2010 [75]
	NCT00258700	Phase III	UK, Poland	Infants	2, 3, 4 months 12 months	500 476	Pace et al., 2007 [69] Pace et al., 2008 [73] Khatami et al., 2011 [76]
	NCT00334334 NCT00463437	Phase III	Germany, Poland, Spain	Infants	2, 4, 6 months 11–18 months	1548 1437	Wysocki et al., 2009 [80] Knuf et al., 2009 [71]
	NCT00326118	Phase III	Australia	Toddlers	12–18 months	433	Booy et al., 2011 [72]
	ISRCTN72858898	Phase IV	UK	Children	6–12 years	249	Perrett et al., 2010 [77]

	NCT00127855	Phase II	Australia	Infants	2, 4, 6 months PS challenge at 11–14 months	407 394	Nolan et al., 2007 [81]
	NCT00129116	Phase II	Belgium, Germany	Infants	2, 3, 4 months 12–18 months	388 221	Habermehl et al., 2010 [68]
Hib-MenCY-TT	NCT00129129	Phase II	US	Infants	2, 4, 6 months 12–15 months	756 498	Marchant et al., 2010 [82] Marshall et al., 2010 [83]
	NCT00134719	Phase II	Australia	Infants	2, 4, 6 months 12–15 months	1103 1037	Nolan et al., 2011 [84]
	NCT00289783	Phase III	US, Mexico, Australia	Infants	2, 4, 6 months 12–15 months	4180	Bryant et al., 2011 [85]

	NCT00196950 NCT00126945	Phase II	Belgium, Denmark	Adolescents Adults	15–25 years	175	Østergaard et al., 2009 [86]
	NCT00126984	Phase II	Germany, Austria	Toddlers Children	12–14 months or 3–5 years	508	Knuf et al., 2010 [87]
MenACWY-TT	NCT00464815 NCT00453986	Phase III	Philippines, India, Taiwan + Philippines, Lebanon	Adolescents Adults	11–17 years and 18–55 years	2272^†^	Bermal et al., 2011 [88]
	NCT00454909	Phase II	US	Adolescents Adults	11–25 years	872	Baxter et al., 2011 [89]
	NCT00474266	Phase III	Finland	Toddlers	12–23 months	1000	Vesikari et al., 2011 [90]
	NCT00508261	Phase III	Austria, Germany, Greece	Toddlers	12–23 months	793	Knuf et al., 2011 [91]

*N*: Total vaccinated cohort including subjects who received the study vaccine and control vaccines, NCT: Clinical Trial Registry Numbers, ISRCTN: International Standard Randomized Controlled Trial Number, *Number of the publication in the reference list, ^†^1025 subjects from study NCT00464815 (Philippines, India, Taiwan) and 1247 from study NCT00453986 (Philippines, Lebanon), For study NCT00453986 only safety data are summarised here.

**Table 2:** rSBA-MenC antibody response after Hib-MenC-TT vaccination.

Reference	Primary vaccination	Booster vaccination	Timepoint	*N*	rSBA-MenC ≥1 : 8		rSBA-MenC ≥1 : 128		GMT	
					%	[95% CI]	%	[95% CI]	Value	[95% CI]
Schmitt et al., 2007 [70] 2, 3, 4 + 12–15 months of age	Hib-MenC-TT + DTPa-HBV-IPV	Men-PS + DTPa-HBV-IPV/Hib	PD3	95	100	[96.2–100]	—	—	944.2	[779.4–1143.8]
			Pre-Ch	90	87.1	[78.0–93.4]	—	—	159.3	[107.9–235.3]
			Post-Ch	83	100	[95.5–100]	—	—	5385.4	[4425.0–5554.2]
	MenC-CRM_197_ + DTPa-HBV-IPV/Hib	Men-PS + DTPa-HBV-IPV/Hib	PD3	105	100	[96.5–100]	—	—	1400.7	[1165.4–1683.5]
			Pre-Ch	93	80.2	[70.6–87.8]	—	—	104.0	[68.4–158.3]
			Post-Ch	86	96.5	[90.0–99.3]	—	—	1552.6	[1044.4–2307.9]

Pace et al., 2007 & 2008 [69, 73] Khatami et al., 2011 [76] 2, 3, 4 + 12–15 months of age	Hib-MenC-TT + DTPa-IPV	Hib-MenC-TT + MMR	PD3	354	99.2	[97.5–99.8]	92.9	[89.8–95.4]	581.1	[514.7–656.2]
			Pre-B	346	78.0	[73.3–82.3]	43.9	[38.6–49.3]	61.3	[50.9–73.7]
			Post-B	347	99.1	[97.5–99.8]	97.7	[95.5–99.0]	2193.7	[1881.1–2558.1]
			Post-B (Y1)	200	89.0	[83.8–93.0]	54.5	[47.3–61.5]	123.0	[98.9–153.0]
			Post-B (Y2)	219	67.1	[60.5–73.3]	39.3	[32.8–46.1]	48.0	[36.8–62.6]
	MenC-CRM_197_ + DTPa-IPV/Hib	Hib-MenC-TT + MMR	PD3	117	100	[96.9–100]	91.1	[95.3–100]	1002.6	[833.8–1205.6]
			Pre-B	109	67.9	[58.3–76.5]	33.0	[24.3–42.7]	38.6	[27.5–54.2]
			Post-B	114	95.6	[90.1–98.6]	86.0	[78.2–91.8]	477.9	[357.3–639.2]
			Post-B (Y1)	59	69.5	[56.1–80.8]	28.8	[17.8–42.1]	35.7	[23.4–54.5]
			Post-B (Y2)	74	40.5	[29.3–52.6]	13.5	[6.7–23.5]	14.4	[9.7–21.6]

Tejedor et al., 2007 & 2008 [62, 74] 2, 4, 6 + 13–14 months of age	Hib-MenC-TT + DTPa-HBV-IPV	Hib-MenC-TT	PD3	111	100	[96.7–100]	99.1	95.1–100	2467.1	[2045.7–2975.3]
			Pre-B	81	96.3	[89.6–99.2]	84.0	74.1–91.2	366.1	[270.1–496.2]
			Post-B	81	100	[95.5–100]	100	[95.5–100]	5266.2	[4265.6–6501.3]
			Post-B (Y1)	56	87.5	[75.9– 94.8]	—	—	94.0	[59.8–147.9]
	MenC-TT + DTPa/Hib-containing	Hib-MenC-TT	PD2	107	100	[96.6–100]	98.1	[93.4–99.8]	1542.9	[1282.2–1856.5]
			Pre-B	169	90.5	[85.1–94.5]	59.8	[52.0–67.2]	131.0	[103.5–165.6]
			Post-B	167	99.4	[96.7–100]	99.4	[96.7–100]	11710.0	[9441.5–14524.8]
			Post-B (Y1)	121	95.0	[89.5–98.2]	—	—	293.8	[212.8–405.7]
	MenC-CRM_197_ + DTPa-HBV-IPV/Hib	DTPa-HBV-IPV/Hib	PD3	114	99.1	[95.2–100]	98.2	93.8–99.8	1833.7	[1493.7–2251.0]
			Pre-B	82	85.4	[75.8–92.2]	56.1	44.7–67.0	120.5	[80.2–180.9]
			Post-B	84	77.4	[67.0–85.8]	56.0	[44.7–66.8]	94.1	[59.6–148.7]

Carmona et al., 2010 [75] 2, 4, 6 + 13–14 months of age	MenC-CRM_197_ + DTPa/Hib-containing	Hib-MenC-TT + MMR	Pre-B*	93	88.2	[79.8–93.9]	46.2	[35.8–56.9]	103.8	[74.2–145.1]
			Post-B	95	98.9	[94.3–100]	89.5	[81.5–94.8]	670.2	[497.9–902.1]
	MenC-CRM_197_ + DTPa/Hib-containing	Hib-MenC-TT	Pre-B*	94	85.1	[76.3–91.6]	56.4	[45.8–66.6]	107.1	[74.1–154.7]
			Post-B	95	98.9	[94.3–100]	92.6	[85.4–97.0]	685.0	[527.0–890.4]

Habermehl et al., 2010 [68] 2, 3, 4 + 12–18 months	Hib-MenC-TT + DTPa-HBV-IPV	Hib-MenC-TT + DTPa-HBV-IPV	PD3	74	100	[95.1–100]	95.9	[88.6–99.2]	871.0	[677.3–1120.0]
			Pre-B*	41	95.1	[83.5–99.4]	65.9	[49.4–79.9]	150.6	[96.7–234.5]
			Post-B	41	100	[91.4–100]	97.6	[87.1–99.9]	3226.2	[2294.7–4535.8]
	MenC-CRM_197_ + DTPa-HBV-IPV/Hib	MenC-CRM_197_ + DTPa-HBV-IPV/Hib	PD3	71	100	[94.9–100]	100	[94.9–100]	3557.6	[2978.8–4248.8]
			Pre-B*	38	94.7	[82.3–99.4]	68.4	[51.3–82.5]	195.5	[122.4–312.2]
			Post-B	39	100	[91.0–100]	100	[91.0–100]	11819.3	[8458.6–16515.2]

Knuf et al., 2009 [71] 2, 4, 6 + 11–18 months of age	Hib-MenC-TT + PHiD-CV + DTPa-HBV-IPV	Hib-MenC-TT + PHiD-CV + DTPa-HBV-IPV	PD3	137	100	[97.3–100]	97.1	[92.7–99.2]	1590.9	[1298.5–1949.1]
			Post-B	79	100	[95.4–100]	100	[95.4–100]	5099.2	[3940.4–6598.9]
	Hib-MenC-TT + 7vCRM + DTPa-HBV-IPV	Hib-MenC-TT + 7vCRM + DTPa-HBV-IPV	PD3	120	99.2	[95.4–100]	95.0	[89.4–98.1]	1207.7	[964.4–1512.3]
			Post-B	76	100	[95.3–100]	97.4	[90.8–99.7]	3269.8	[2489.7–4294.4]
	MenC-TT + DTPa-HBV-IPV/Hib + PHiD-CV	MenC-TT + DTPa/Hib-containing + PHiD-CV	PD2	177	100	[97.9–100]	97.2	[93.5–99.1]	1474.2	[1263.3–1 720.4]
			Post-B	84	100	[95.7–100]	100	[95.7–100]	4587.8	[3763.1–5593.2]
	MenC-CRM_197_ + DTPa-HBV-IPV/Hib + PHiD-CV	MenC-CRM_197_ + DTPa/Hib-containing + PHiD-CV	PD2	165	98.8	[95.7–99.9]	97.0	[93.1–99.0]	1299.8	[1082.0–1561.5]
			Post-B	89	100	[95.9–100]	98.9	[93.9–100]	2779.6	[2198.5–3514.2]

Booy et al., 2011 [72]12–18 months	3xDTPa/Hib-TT or2xHib-OMP	Hib-MenC-TT + MMR	Pre-Vac	255	14.5	[10.4–19.4]	5.9	[3.3–9.5]	6.3	[5.5–7.3]
			Post-Vac	281	99.6	[98.0–100]	87.9	[83.5–91.5]	482.8	[420.7–554.2]
			Post-Vac (Y1)	249	86.7	[81.9–90.7]	47.0	[40.7–53.4]	91.7	[75.6–111.3]
	3xDTPa/Hib-TT or 2xHib-OMP	Hib-TT + MenC-CRM_197_ + MMR	Pre-Vac	83	8.4	[3.5–16.6]	3.6	[0.8–10.2]	5.5	[4.3–7.2]
			Post-Vac	98	100	[96.3–100]	90.8	[83.3–95.7]	621.0	[480.3–802.9]
			Post-Vac (Y1)	89	76.4	[66.2–84.8]	41.6	[31.2–52.5]	63.8	[43.3–94.1]

*N*: numbers of subjects with available data, %: percentage of subjects with titre within the specified range, 95% CI: 95% confidence interval, GMT: Geometric Mean Titre, PD2: one month after dose 2 (Primary ATP cohort for immunogenicity), PD3: one month after dose 3 (Primary ATP Cohort for Immunogenicity), Pre-Ch: just prior to meningococcal polysaccharide challenge (ATP Cohort for Persistence), Post-Ch: one month after meningococcal polysaccharide challenge (ATP Cohort for Immunogenicity), Pre-B: just prior to the booster vaccination (ATP Cohort for Persistence), Pre-B*: just prior to the booster vaccination (ATP Cohort for Immunogenicity), Post-B: one month after booster vaccination (Booster ATP Cohort for Immunogenicity), Post-B (Y1): one year after booster vaccination (ATP Cohort for Persistence Year 1), Post-B (Y2): two years after booster vaccination (ATP Cohort for Persistence Year 1), Pre-Vac: just prior to the vaccination (Vaccination ATP cohort for Immunogenicity), Post-Vac: one month after vaccination (Vaccination ATP cohort for Immunogenicity), Post-Vac (Y1): one year after vaccination (ATP cohort for Persistence Year 1), Results of this table were previously published [62, 68–76].

**Table 3:** rSBA-MenC, rSBA-MenY antibody responses after Hib-MenCY-TT vaccination.

Reference	Vaccine dose 1–3	Vaccine dose 4	Timepoint	*N*	rSBA ≥ 1 : 8		rSBA ≥ 1 : 128		GMT	
					%	[95% CI]	%	[95% CI]	Value	[95% CI]
rSBA-MenC										

Habermehl et al., 2010 [68] 2, 3, 4 + 12–18 months of age	Hib-MenCY-TT + DTPa-HBV-IPV	Hib-MenCY-TT + DTPa-HBV-IPV	PD3	70	100	[94.9–100]	95.7	[88.0–99.1]	1005.8	[773.5–1308.0]
			Pre-D4	46	91.3	[79.2–97.6]	63.0	[47.5–76.8]	153.1	[90.4–259.4]
			PD4	40	100	[91.2–100]	100	[91.2–100]	4762.6	[3427.5–6617.7]
	MenC-CRM_197_ + DTPa-HBV-IPV/Hib	MenC-CRM_197_ + DTPa-HBV-IPV/Hib	PD3	71	100	[94.9–100]	100	[94.9–100]	3557.6	[2978.8–4248.8]
			Pre-D4	42	95.2	[83.8–99.4]	69.0	[52.9–82.4]	199.2	[129.6–306.2]
			PD4	39	100	[91.0–100]	100	[91.0–100]	11819.3	[8458.6–16515.2]

Nolan et al., 2007 [81] 2, 4, 6 + 11–14 months of age	Hib-MenCY-TT + DTPa-HBV-IPV	Men-PS Challenge	PD3	74*	100	[94.8–100]	98.6	[92.2–100]	1293.1	[1027.7–1627.1]
			Pre-Ch	80*	97.4	[90.8–99.7]	78.9	[68.1–87.5]	265.5	[197.8–356.3]
			Post-Ch	73*	100	[94.8–100]	98.6	[92.2–100]	1985.5	[1542.6–2555.8]
	MenC-CRM_197_ + DTPa-HBV-IPV/Hib	Men-PS Challenge	PD3	77*	100	[95.1–100]	98.6	[92.7–100]	1931.9	[1541.2–2421.6]
			Pre-Ch	81*	90.8	[81.9–96.2]	64.5	[52.7–75.1]	176.6	[117.9–264.4]
			Post-Ch	79*	97.5	[91.2–99.7]	84.8	[75.0–91.9]	774.8	[536.7–1118.5]

Nolan et al., 2011 [84] 2, 4, 6 + 12–15 months of age	Hib-MenCY-TT + 7vCRM + DTPa-HBV-IPV	Hib-MenCY-TT + MMR + VAR	PD3	287	99.0	[97.0–99.8]	94.4	[91.1–96.8]	805	[700–925]
			Pre-D4	495	90.7	[87.8–93.1]	48.7	[44.2–53.2]	103	[90.1–117]
			PD4	496	99.6	[98.6–100]	97.2	[95.3–98.4]	1697	[1516–1900]
	MenC-CRM_197_ + Hib-TT + 7vCRM + DTPa-HBV-IPV	Hib-MenCY-TT + MMR + VAR	PD3	99	100	[96.3–100]	96.0	[90.0–98.9]	790	[649–961]
			Pre-D4	169	84.6	[78.3–89.7]	29.6	[22.8–37.1]	53.7	[43.0–67.1]
			PD4	162	95.7	[91.3–98.2]	71.0	[63.3–77.8]	262	[204–336]

rSBA-MenY										

Habermehl et al., 2010 [68] 2, 3, 4 + 12–18 months of age	Hib-MenCY-TT + DTPa-HBV-IPV	Hib-MenCY-TT + DTPa-HBV-IPV	PD3	69	97.1	[89.9–99.6]	92.8	[83.9–97.6]	470.7	[351.1–631.2]
			Pre-D4	45	86.7	[73.2–94.9]	60.0	[44.3–74.3]	103.2	[64.6–164.9]
			PD4	40	100	[91.2–100]	100	[91.2–100]	1708.1	[1313.3–2221.4]

Nolan et al., 2007 [81] 2, 4, 6 + 11–14 months of age	Hib-MenCY-TT + DTPa-HBV-IPV	Men-PS Challenge	PD3	74*	98.5	[92.0–100]	95.5	[87.5–99.1]	843.5	[640.1–1111.7]
			Pre-Ch	80*	89.2	[79.8–95.2]	60.8	[48.8–72.0]	114.7	[80.0–164.4]
			Post-Ch	73*	100	[94.7–100]	100	[94.7–100]	1838.0	[1427.9–2366.0]

Nolan et al., 2011 [84] 2, 4, 6 + 12–15 months of age	Hib-MenCY-TT + 7vCRM + DTPa-HBV-IPV	Hib-MenCY-TT + MMR + VAR	PD3	288	99.7	[98.1–100]	91.7	[87.9–94.6]	728	[636–835]
			Pre-D4	514	98.4	[97.0–99.3]	80.5	[76.9–83.9]	264	[241–290]
			PD4	496	100	[99.3–100]	99.4	[98.2–99.9]	1987	[1826–2161]

*N*: numbers of subjects with available data, *N: numbers of subjects in the ATP Cohort, %: percentage of subjects with titre within the specified range, 95% CI: 95% confidence interval, GMT: Geometric Mean Titre, PD3: one month after dose 3 (ATP Cohort for Immunogenicity), Pre-D4: just prior dose 4 (ATP Cohort for Persistence), PD4: one month after dose 4 (ATP Cohort for Immunogenicity), Pre-Ch: just prior to meningococcal polysaccharide challenge (ATP Cohort for safety challenge phase), Post-Ch: one month after meningococcal polysaccharide challenge (ATP Cohort for Immunogenicity), Results of this table were previously published [68, 81, 84].

**Table 4:** hSBA-MenC, hSBA-MenY antibody responses after Hib-MenCY-TT vaccination.

Reference	Vaccine dose 1–3	Vaccine dose 4	Timepoint	*N*	hSBA ≥ 1 : 4		hSBA ≥ 1 : 8		GMT	
					%	[95% CI]	%	[95% CI]	Value	[95% CI]
hSBA-MenC										

Marchant et al., 2010 [82] & Marshall et al., 2010 [83] 2, 4, 6 + 12–15 months of age	Hib-MenCY-TT + 7vCRM + DTPa-HBV-IPV	Hib-MenCY-TT + 7vCRM	PD3	121	97.5	[92.9–99.5]	95.9	[90.6–98.6]	523.0	[398.8–686.0]
			Pre-D4	63	90.5	[80.4–96.4]	90.5	[80.4–96.4]	71.8	[48.0–107.5]
			PD4	65	96.9	[89.3–99.6]	96.9	[89.3–99.6]	657.1	[438.4–984.8]
			PD4 (Y1)	116	96.6	[91.4–99.1]	96.6	[91.4–99.1]	150.1	[116.5–193.4]
	Hib-TT + 7vCRM + DTPa-HBV-IPV	Hib-MenCY-TT + 7vCRM	PD4	35	94.3	[80.8–99.3]	94.3	[80.8–99.3]	72.5	[46.4–113.3]
			PD4 (Y1)	48	70.8	[55.9–83.0]	70.8	[55.9–83.0]	26.3	[14.8–46.5]

Nolan et al., 2011 [84] 2, 4, 6 + 12–15 months of age	Hib-MenCY-TT + 7vCRM + DTPa-HBV-IPV	Hib-MenCY-TT + MMR + VAR	PD3	83	100	[95.7–100]	100	[95.7–100]	379	[295–489]
			Pre-D4	134	93.3	[87.6–96.9]	93.3	[87.6–96.9]	97.7	[74.0–129]
			PD4	132	99.2	[95.9–100]	99.2	[95.9–100]	1808	[1398–2337]
	MenC-CRM_197_ + Hib-TT + 7vCRM DTPa-HBV-IPV	Hib-MenCY-TT + MMR + VAR	PD3	28	100	[87.7–100]	100	[87.7–100]	254	[193–335]
			Pre-D4	54	85.2	[72.9–93.4]	85.2	[72.9–93.4]	34.6	[22.8–52.6]
			PD4	43	95.3	[84.2–99.4]	95.3	[84.2–99.4]	172	[94.7–311]

Bryant et al., 2011 [85] 2, 4, 6 + 12–15 months of age	Hib-MenCY-TT + DTPa-HBV-IPV + 7vCRM (Mexico)	Hib-MenCY-TT + MMR + VAR	PD3	134	99.3	[95.9–100]	99.3	[95.9–100]	3172.6	[2657.9–3786.8]
			PD4	39	100	[91.0–100]	100	[91.0–100]	10132.9	[8008.0–12821.7]
	Hib-MenCY-TT + DTPa-HBV-IPV + 7vCRM (US)	Hib-MenCY-TT + MMR + VAR	PD3	491	98.8	[97.4–99.6]	98.8	[97.4–99.6]	967.6	[864.0–1083.5]
			Pre-D4	420	96.0	[93.6–97.6]	96.0	[93.6–97.6]	176.5	[153.9–202.4]
			PD4	331	98.5	[96.5– 99.5]	98.5	[96.5– 99.5]	2039.8	[1746.3–2382.6]

hSBA-MenY										

Marchant et al., 2010 [82] & Marshall et al., 2010 [83] 2, 4, 6 + 12–15 months of age	Hib-MenCY-TT + 7vCRM + DTPa-HBV-IPV	Hib-MenCY-TT + 7vCRM	PD3	141	91.5	[85.6–95.5]	89.4	[83.1–93.9]	139.8	[102.6–190.5]
			Pre-D4	66	54.5	[41.8–66.9]	50.0	[37.4–62.6]	12.2	[7.7–19.1]
			PD4	65	95.4	[87.1–99.0]	95.4	[87.1–99.0]	246.6	[168.0–362.1]
			PD4 (Y1)	105	84.8	[76.4–91.0]	83.8	[75.3–90.3]	128.8	[86.2–192.5]
	Hib-TT + 7vCRM + DTPa-HBV-IPV	Hib-MenCY-TT + 7vCRM	PD4	35	60.0	[42.1–76.1]	57.1	[39.4–73.7]	11.1	[6.5–19.0]
			PD4 (Y1)	47	66.0	[50.7–79.1]	66.0	[50.7–79.1]	41.1	[20.4–82.9]

Nolan et al., 2011 [84] 2, 4, 6 + 12–15 months of age	Hib-MenCY-TT + 7vCRM + DTPa-HBV-IPV	Hib-MenCY-TT + MMR + VAR	PD3	85	100	[95.8–100]	100	[95.8–100]	86.4	[68.9–108.3]
			Pre-D4	114	91.2	[84.5–95.7]	90.4	[83.4–95.1]	81.2	[58.6–112.6]
			PD4	122	97.5	[93.0–99.5]	97.5	[93.0–99.5]	1002	[741–1356]

Bryant et al., 2011 [85] 2, 4, 6 + 12–15 months of age	Hib-MenCY-TT + DTPa-HBV-IPV + 7vCRM (Mexico)	Hib-MenCY-TT + MMR + VAR	PD3	135	99.3	[95.9–100]	99.3	[95.9–100]	837.2	[696.4–1006.3]
			PD4	40	100	[91.2–100]	100	[91.2–100]	5775.8	[4488.9–7431.7]
								
	Hib-MenCY-TT + DTPa-HBV-IPV + 7vCRM (US)	Hib-MenCY-TT + MMR + VAR	PD3	481	96.3	[94.2–97.8]	95.8	[93.7–97.4]	236.6	[205.7–272.1]
			Pre-D4	419	93.6	[90.8–95.7]	92.8	[89.9–95.1]	117.5	[101.3–136.2]
			PD4	342	98.8	[97.0–99.7]	98.8	[97.0–99.7]	1389.5	[1205.0–1602.2]

*N*: numbers of subjects with available data, 95% CI: 95% confidence interval, GMT: Geometric Mean Titre, %: percentage of subjects with titre within the specified range, PD3: one month after dose 3 (ATP Cohort for Immunogenicity), Pre-D4: just prior dose 4 (ATP Cohort for Persistence), PD4: one month after dose 4 (ATP Cohort for Immunogenicity), PD4 (Y1): one year after dose 4 (ATP cohort for Persistence Year 1), Results of this table were previously published [82–85].

**Table 5:** rSBA-MenA, rSBA-MenC, rSBA-MenW-135, and rSBA-MenY antibody response one month [87, 91] or 42 days [90] after one dose of MenACWY-TT in toddlers (ATP immunogenicity cohort).

Reference	Age group	Vaccine	*N*	%≥1 : 8 [95% CI]	%≥1 : 128 [95% CI]	GMT [95% CI]
rSBA-MenA						

Knuf et al., 2010 [87]	12–14 months	MenACWY-TT	42	100 [91.6–100]	100 [91.6–100]	5665.2 [4086.0–7854.9]
Vesikari et al., 2011 [90]	12–23 months	MenACWY-TT	354	99.7 [98.4–100]	99.7 [98.4–100]	2205.0 [2007.8–2421.6]
		MenACWY-TT + MMRV	360	100 [99.0–100]	99.7 [98.5–100]	2085.9 [1905.3–2283.6]
Knuf et al., 2011 [91]	12–23 months	MenACWY-TT	183	98.4 [95.3–99.7]	97.8 [94.5–99.4]	3169.9 [2577.2–3898.8]
		MenACWY-TT*	178	100 [97.9–100]	100 [97.9–100]	1938.3 [1699.1–2211.2]
		MenACWY-TT + DTPa-HBV-IPV/Hib	193	100 [98.1–100]	100 [98.1–100]	3152.9 [2752.5–3611.4]

rSBA-MenC						

Knuf et al., 2010 [87]	12–14 months	MenACWY-TT	42	100 [91.6–100]	95.2 [83.8–99.4]	983.5 [718.7–1345.9]
		MenC-CRM_197_	46	97.8 [88.5–100]	87.0 [73.7–95.1]	372.5 [257.7–538.4]
Vesikari et al., 2011 [90]	12–23 months	MenACWY-TT	354	99.7 [98.4–100]	95.8 [93.1–97.6]	477.6 [437.3–521.6]
		MenACWY-TT + MMRV	357	100 [99.0–100]	94.4 [91.5–96.5]	519.0 [470.9–571.9]
		MenC-CRM_197_	121	97.5 [92.9–99.5]	70.2 [61.3–78.2]	212.3 [170.0–265.2]
Knuf et al., 2011 [91]	12–23 months	MenACWY-TT	183	97.3 [93.7–99.1]	94.0 [89.5–97.0]	828.7 [672.4–1021.4]
		MenACWY-TT*	178	100 [97.9–100]	88.2 [82.5–92.5]	386.0 [333.9–446.2]
		MenACWY-TT + DTPa-HBV-IPV/Hib	191	100 [98.1–100]	99.0 [96.3–99.9]	879.7 [763.1–1014.0]
		MenC-CRM_197_	114	98.2 [93.8–99.8]	89.5 [82.3–94.4]	691.4 [520.8–917.9]

rSBA-MenW-135						

Knuf et al., 2010 [87]	12–14 months	MenACWY-TT	43	100 [91.8–100]	100 [91.8–100]	3975.2 [3065.7–5154.5]
Vesikari et al., 2011 [90]	12–23 months	MenACWY-TT	354	100 [99.0–100]	99.4 [98.0–99.9]	2681.7 [2453.1–2931.6]
		MenACWY-TT + MMRV	360	100 [99.0–100]	100 [99.0–100]	2055.8 [1871.0–2258.9]
Knuf et al., 2011 [91]	12–23 months	MenACWY-TT	186	98.4 [95.4–99.7]	96.8 [93.1–98.8]	4022.3 [3269.2–4948.8]
		MenACWY-TT*	179	100 [98.0–100]	99.4 [96.9–100]	2466.4 [2175.4–2796.4]
		MenACWY-TT + DTPa-HBV-IPV/Hib	193	100 [98.1–100]	100 [98.1–100]	4147.0 [3670.1–4685.8]

rSBA-MenY						

Knuf et al., 2010 [87]	12–14 months	MenACWY-TT	42	100 [91.6–100]	100 [91.6–100]	2295.1 [1701.5–3095.8]
Vesikari et al., 2011 [90]	12–23 months	MenACWY-TT	354	100 [99.0–100]	99.7 [98.4–100]	2729.4 [2472.7–3012.8]
		MenACWY-TT + MMRV	359	100 [99.0–100]	99.7 [98.5–100]	2282.4 [2051.3–2539.5]
Knuf et al., 2011 [91]	12–23 months	MenACWY-TT	185	97.3 [93.8–99.1]	96.2 [92.4–98.5]	3167.7 [2521.9–3978.9]
		MenACWY-TT*	179	99.4 [96.9–100]	99.4 [96.9–100]	2446.9 [2088.5–2866.8]
		MenACWY-TT + DTPa-HBV-IPV/Hib	192	100 [98.1–100]	100 [98.1–100]	3461.8 [2990.1–4007.9]

*N*: numbers of subjects with available data, %: percentage of subjects with titre within the specified range, 95% CI: 95% confidence interval, GMT: Geometric Mean Titre, *MenACWY-TT given one month after DTPa-HBV-IPV/Hib vaccine, Results of this table were previously published [87, 90, 91].

**Table 6:** rSBA-MenA, rSBA-MenC, rSBA-MenW-135, and rSBA-MenY antibody response one month after MenACWY-TT vaccination in children and adolescents (ATP immunogenicity cohort).

Reference	Age group	Vaccine	*N*	VRR	[95% CI]	*N*	GMT	[95% CI]
rSBA-MenA								

Østergaard et al., 2009 [86]	15–19 years	MenACWY-TT	23	87.0	[66.4–97.2]	24	9264	[6342–13532]
		ACWY-PS	23	78.3	[56.3–92.5]	25	8284	[6035–11370]
Knuf et al., 2010 [87]	3–5 years	MenACWY-TT	40	92.5	[79.6–98.4]	49	8299.4	[6734.2–10228.4]
		ACWY-PS	31	80.6	[62.5–92.5]	33	3798.4	[2888.9–4994.2]
Bermal et al., 2011 [88]	11–17 years	MenACWY-TT	615	85.4	[82.3–88.1]	752	6106.8	[5739.5–6497.6]
		ACWY-PS	215	79.5	[73.5–84.7]	252	3203.0	[2854.1–3594.6]

rSBA-MenC								

Østergaard et al., 2009 [86]	15–19 years	MenACWY-TT	24	95.8	[78.9–99.9]	24	4329	[2404–7795]
		ACWY-PS	24	91.7	[73.0–99.0]	25	1567	[757–3245]
Knuf et al., 2010 [87]	3–5 years	MenACWY-TT	47	93.6	[82.5–98.7]	49	1577.8	[1123.5–2215.9]
		ACWY-PS	32	81.3	[63.6–92.8]	34	445.4	[263.3–753.3]
Bermal et al., 2011 [88]	11–17 years	MenACWY-TT	719	97.1	[95.6–98.2]	754	12645.5	[11531.8–13866.7]
		ACWY-PS	237	96.6	[93.5–98.5]	252	8271.6	[6937.3–9862.4]

rSBA-MenW-135								

Østergaard et al., 2009 [86]	15–19 years	MenACWY-TT	23	91.3	[72.0–98.9]	24	4422	[2939–6653]
		ACWY-PS	25	96.0	[79.6–99.9]	25	3486	[2447–4967]
Knuf et al., 2010 [87]	3–5 years	MenACWY-TT	47	100	[92.5–100]	49	5987.2	[4846.9–7395.7]
		ACWY-PS	32	96.9	[83.8–99.9]	34	1811.1	[1233.3–2659.5]
Bermal et al., 2011 [88]	11–17 years	MenACWY-TT	717	96.5	[94.9–97.7]	759	8390.1	[7777.8–9050.7]
		CWY-PS	242	88.0	[83.2–91.8]	252	2679.3	[2363.7–3037.2]

rSBA-MenY								

Østergaard et al., 2009 [86]	15–19 years	MenACWY-TT	24	79.2	[57.8–92.9]	24	2756	[2023–3755]
		ACWY-PS	25	88.0	[68.8–97.5]	25	3056	[1892–4937]
Knuf et al., 2010 [87]	3–5 years	MenACWY-TT	48	100	[92.6–100]	49	6433.0	[4921.0–8409.6]
		ACWY-PS	34	79.4	[62.1–91.3]	34	1435.5	[972.6–2118.7]
Bermal et al., 2011 [88]	11–17 years	MenACWY-TT	737	93.1	[91.0–94.8]	758	13865.2	[12968.1–14824.4]
		ACWY-PS	246	78.0	[72.3–83.1]	252	5245.3	[4644.2–5924.1]

*N*: numbers of subjects with available data, 95% CI: 95% confidence interval, GMT: geometric mean titre, VRR: vaccine response rate, Vaccine response defined as: (1) for initially seronegative subjects, antibody titres ≥1 : 32 at one month after vaccination, (2) for initially seropositive subjects, antibody titres at one month after vaccination ≥4-fold the pre-vaccination antibody titre, Results of this table were previously published [86–88].

**Table 7:** Percentage of subjects with hSBA titres ≥1 : 8 and hSBA GMTs against serogroups A, C, W-135, and Y at 42 days after one dose of MenACWY-TT in toddlers (ATP immunogenicity cohort).

Vaccine	*N*	% ≥ 1 : 8 [95% CI]	GMT [95% CI]
hSBA-MenA			

MenACWY-TT	338	77.2 [72.4–81.6]	19.0 [16.4–22.1]
MenACWY-TT + MMRV	348	83.9 [79.6–87.6]	33.7 [28.9–39.2]

hSBA-MenC			

MenACWY-TT	341	98.5 [96.6–99.5]	196.0 [175.4–219.0]
MenACWY-TT + MMRV	346	98.0 [95.9–99.2]	209.1 [183.8–238.0]
MenC-CRM_197_	116	81.9 [73.7–88.4]	40.3 [29.5–55.1]

hSBA-MenW-135			

MenACWY-TT	336	87.5 [83.5–90.8]	48.9 [41.2–58.0]
MenACWY-TT + MMRV	337	82.8 [78.3–86.7]	57.3 [47.0–69.9]

hSBA-MenY			

MenACWY-TT	329	79.3 [74.5–83.6]	30.9 [25.8–37.1]
MenACWY-TT + MMRV	333	81.4 [76.8–85.4]	38.7 [32.2–46.7]

*N*: numbers of subjects with available data, %: percentage of subjects with titre within the specified range, 95% CI: 95% confidence interval, GMT: Geometric Mean Titre, Results of this table were previously published [90].
